# Connectome Analysis of Brain Functional Network Alterations in Depressive Patients with Suicidal Attempt

**DOI:** 10.3390/jcm8111966

**Published:** 2019-11-14

**Authors:** Jun-Cheng Weng, Yu-Syuan Chou, Yuan-Hsiung Tsai, Chun-Te Lee, Ming-Hong Hsieh, Vincent Chin-Hung Chen

**Affiliations:** 1 Department of Medical Imaging and Radiological Sciences, Chang Gung University, Taoyuan 33302, Taiwan; jcweng@mail.cgu.edu.tw; 2 Medical Imaging Research Center, Institute for Radiological Research, Chang Gung University and Chang Gung Memorial Hospital at Linkou, Taoyuan 33302, Taiwan; 3 Department of Psychiatry, Chang Gung Memorial Hospital, Chiayi 61363, Taiwan; 4 Department of Medical Imaging and Radiological Sciences, Chung Shan Medical University, Taichung 40201, Taiwan; ttt90243@yahoo.com.tw; 5 School of Medicine, Chang Gung University, Taoyuan 33302, Taiwan; russell.tsai@gmail.com; 6 Department of Diagnostic Radiology, Chang Gung Memorial Hospital, Chiayi 61363, Taiwan; 7 Department of Psychiatry, School of Medicine, Chung Shan Medical University and Hospital, Taichung 40201, Taiwan; lee0606@ms24.hinet.net (C.-T.L.); mhhpsy@hotmail.com (M.-H.H.)

**Keywords:** suicide attempt, resting-state functional connectivity, graph theoretical analysis, network-based statistical analysis

## Abstract

Our study aimed to clarify the neuroimaging correlates of suicide attempt by comparing differences in functional magnetic resonance imaging (fMRI) among depressed suicide attempters, depressed patients without suicide attempt history, and healthy controls through comprehensive and novel fMRI analyses and methods in the same study population. The association between depression severity and aspects of the brain imaging was also discussed. Our study recruited 109 participants who were assigned to three groups: 33 depressed patients with suicide attempt (SA), 32 depressed patients without suicide attempt (NS), and 44 healthy controls (HC). All participants were scanned using a 3 T MRI imaging system to obtain resting-state functional images. In seed-based correlation analysis, we found altered functional connectivity in some brain regions of the SA compared with the NS or HC, especially in the hippocampus and thalamus. In the voxel-based analysis, our results showed differential activation and regional homogeneity of the temporal lobe and several brain regions in the SA compared with the NS and HC. We also found that some brain areas correlated with the Hamilton Depression Rating Scale (HAM-D), anxiety, and depression scores, especially in the frontal and temporal lobes. Graph theoretical analysis (GTA) and network-based statistical (NBS) analyses revealed different topological organization as well as slightly better global integration and worse local segregation of the brain network (i.e., more like a random network) in depressed participants compared with healthy participants. We concluded that the brain function of major depressive disorders with and without suicide attempts changed compared with healthy participants.

## 1. Introduction

Suicide is an important and serious public health problem worldwide. In 2012, approximately 800,000 people died from self-inflicted injury [1]. The strongest predictor of completed suicide is suicide attempt [2]; suicide risk among those with a history of suicide attempt is hundreds of times higher than in the general population [3,4]. The lifetime prevalence of suicide attempt is high in the general population: 4.6% in the National Comorbidity Survey (NCS) [5] and 4.3% in the earlier epidemiologic catchment area survey (ECA) [6].

Suicide risk evaluation still relies on clinicians’ experiences, but many health-related workers are not familiar with suicide risk assessment. Meanwhile, the denial of suicidal ideation can easily become the justification for reduced vigilance among persons with high suicidal potential [7]. Exploring the quantitative indicators that reflect stable changes in brain function is important for creating more effective preventive treatment programs, for better identifying at-risk individuals, and for developing more specific therapeutic tools [8]. Recently, neurobiological studies have provided further evidence for this concept by demonstrating that suicidal behavior is a state-based condition stored in neural circuitry that can be quickly switched on by the recall of an experience of mental pain [9].

Compared to depressed patients without suicide attempt history (NS), depressed patients with a suicide attempt history (SA) showed a number of neurobiological differences including the following: increased resting-state functional connectivity of the left amygdala with the right insula and left superior orbitofrontal area and increased connectivity of the right amygdala with the left middle temporal area [10]; increased connectivity in the left cerebellum and the left lingual gyrus and decreased connectivity in the right precuneus [11]; decreased fractional amplitude of low-frequency fluctuation (ALFF) values in the left superior frontal gyrus medial frontal gyrus [12]; and increased ALFF in the right superior temporal gyrus and decreased ALFF in the right ventral medial frontal gyrus [13]. Compared to healthy controls (HC), SA patients showed a number of differences including the following: increased functional connectivity in select default mode network (DMN) regions and increased connectivity in the left cerebellum and decreased connectivity in the right posterior cingulate cortex, [11]; increased ALFF in the right superior temporal gyrus, left middle temporal gyrus, and left middle occipital gyrus [12]; lower mean regional homogeneity (ReHo) in the left fusiform and supraorbital inferior frontal gyri, left hippocampus, bilateral parahippocampal, and middle frontal gyri, right angular gyrus, and cerebellar lobules RVIII, RII, and LVI. ReHo was higher in the right supraorbital middle frontal gyrus, right inferior parietal lobe, and left precuneus [14]; and increased ALFF in the right superior temporal gyrus, left anterior cingulate cortex and right parahippocampal gyrus and decreased ALFF in the left middle occipital gyrus and left angular gyrus [13].

Previous studies showed a variety of inconsistent findings for the characteristics of people with a history of suicide attempts regarding fMRI results. The problems may be due to experimental limitations of previous studies. These limitations encompass the following: there was no healthy control group [10] or depressed control group [14]; different fMRI analysis methods, such as resting-state functional connectivity [10,11], ALFF [12,13], and ReHo analysis [14]; and low sample size [10,13]. There is also a lack of analysis regarding the associations between depression severity and brain images. Previous studies did not include both healthy and psychiatric control groups, making it impossible to determine which changes are due to the diathesis for suicide attempts and which are due to the primary psychiatric disorders [15].

The present study aimed to clarify the neuroimaging correlates of suicide attempt by comparing the differences in fMRI among the SA patients, NS patients, and healthy controls through a comprehensive and novel method of fMRI analyses in the same study population. The association between depression severity and the brain images was also discussed.

## 2. Materials and Methods

### 2.1. Participants

Our study recruited 109 participants who were assigned into three groups: 33 depressed patients with suicide attempt (hereafter, SA) (age 28–57 years, mean = 43.55 years, standard deviation (SD) = 7.79 years), 32 depressed patients without suicide attempt (NS) (age 21–60 years, mean = 47.88 years, SD = 8.99 years), and 44 healthy controls (HC) (age 20–58 years, mean = 41.98 years, SD = 9.27 years). All participants were at least 20 years of age and right-handed. The participants were recruited via the outpatient clinic of the department of psychiatry at Chiayi Chang Gung Hospital and recruitment advertisements. The confirmation of depressive disorders, suicidal ideation was primarily based on psychiatrists’ diagnosis and the Mini-International Neuropsychiatric Interview (MINI) carried out by the trained research nurse. The final confirmation was carried out by the principle investigator (V.C.H) according to all available information. Exclusion criteria for all participants were any eye diseases (e.g., cataract and glaucoma), history of suicide attempt, another primary severe mental disorder (e.g., schizophrenia or bipolar disorder), alcohol/illicit substance use disorder during the past year, any neurological illnesses, and metallic implants or other contraindications for MRI. The study was approved by the Institutional Review Board of the Chang Gung Memorial Hospital, Chiayi, Taiwan (No. 201602027B0, 104-9337B, 104-0838B). All participants participated in the study after providing informed consent, and all research was performed in accordance with relevant guidelines and regulations. The data that support the findings of this study are available from the first or the corresponding author upon reasonable request.

### 2.2. MRI Data Acquisition

All participants were scanned using a 3 T MRI (Verio, SIEMENS, Erlangen, Germany) imaging system with a standard eight-channel head coil. A gradient-echo echo planar image (EPI) sequence was used to obtain resting-state functional images. The phase-encode direction was along anterior-posterior axis. We asked all subjects to be relaxed, close their eyes, and not think of anything, although they could not fall asleep during the resting-state fMRI scan. The image acquisition parameters were repetition time (TR)/ echo time (TE) = 2000/30 ms, field of view (FOV) = 220 × 220 mm^2^, matrix size = 64 × 64, in-plane resolution (pixel size) = 3.4 × 3.4 mm^2^, thickness = 4 mm, number of repetitions = 300, and 31 axial slices aligned along AC-PC lines without a gap to cover the whole cerebrum.

### 2.3. Functional Image Preprocessing

We used statistical parametric mapping (SPM, Wellcome Department of Cognitive Neurology, London, UK) software to conduct preprocessing of functional images. After slice-timing correction, we calculated the center of mass of each image and realigned the data to the first volume for motion correction (if the result of six head motion parameters exceeded 1 mm translation or 1° rotation, it was excluded from this study). All of the participants met the criteria, and no one was excluded. After motion correction, data were normalized to the standard Montreal Neurological Institute (MNI) space with affine transform, and the data were resampled to isotropic 3-mm voxels. The data were then spatially smoothed using a 6-mm full width at half maximum (FWHM) Gaussian kernel for a better signal-to-noise ratio gain. Then, using the six head motion parameters as covariates, we performed nuisance regression. Then, the whole brain, white matter and cerebrospinal fluid masks were used to remove the physiological noise. Linear detrending and bandpass temporal filtering were performed on the time series of each voxel to minimize the effects of low-frequency drifts and physiological signals by the Resting-State Data Analysis tool kit v1.8 (REST v1.8, Center for Cognition and Brain Disorders, Hangzhou Normal University, Zhejiang, China). Previous studies suggested that the frequencies with important physiological information were in the range of 0.01–0.08 Hz [16,17]. However, some research suggested that complex functional networks may be observed in the range of 0.1–0.12 Hz [18]. Therefore, we extended the frequency range from 0.01 to 0.12 Hz to mitigate the influence of low-frequency drift and high-frequency physiological noise.

### 2.4. Functional Connectivity (FC) and Seed-Based Correlation Analysis (SCA)

As spontaneous, coherent, and low-frequency fluctuations of the blood oxygenation level dependent (BOLD) signal were used for the resting-state analysis. Correlating the averaged BOLD signal of the user-defined region of interest to the BOLD signal of every other single voxel for each participant constructed correlation maps at the voxel level. The SCA analysis of functional connectivity was to locate the seed on the selected ROI coordinates and observe the activation of the contralateral brain region. Our experiments selected brain regions associated with depression, including the amygdala, hippocampus, thalamus, visual cortex, motor cortex, and posterior cingulate cortex, which are connected with the frontal lobe, anterior cingulate cortex and precuneus. To enforce a Gaussian distribution of the correlation data, the Pearson’s correlation r was then transformed into z-scores using the Fisher r to z transformation. 

### 2.5. Amplitude of Low-Frequency Fluctuations (ALFF)

The ALFF was calculated in the frequency range of 0.01–0.12 Hz. The procedure for calculating the ALFF is briefly described as follows: for a given voxel, the time series was first converted to the frequency domain using a fast Fourier transform. The square root of the power spectrum was computed, averaged, and normalized across a predefined frequency interval, which was termed the ALFF at the given voxel [19]. The mean fractional ALFF (mfALFF) can be regarded as a normalized mean ALFF and was calculated using the total energy over the detectable frequency range. The mfALFF can provide a more specific measure of low-frequency oscillatory phenomena than the mALFF [20]. In voxel-based analysis (VBA), we assessed the difference in the mfALFF between the SA, NS, and HC using false discovery rate (FDR)-corrected t-tests. We also probed the relationship between the SA participants’ mfALFF and Hamilton depression rating scale (HAM-D)/Hospital Anxiety and Depression Scale (HADS) scores using multiple regressions. In the study, age, gender and years of education were used as the covariates to eliminate the effect caused by differences between each group. We used a T1-weighted MNI template to create the underlying map to view the results.

### 2.6. Regional Homogeneity (ReHo)

To analyze ReHo, linear detrending and bandpass filtering (0.01–0.12 Hz) were performed on the time series of each voxel by REST v1.8. Evaluating the resting state cortical activity in the SA, NS and HC by using the ReHo approach. Each individual ReHo map was generated by calculating Kendall’s coefficient of concordance (KCC), which computes the ReHo of the BOLD time series data in each voxel and its 26 nearest adjacent voxels [21]. Then, a mask was used to remove non-brain tissues and noise on the ReHo maps, and the individual ReHo maps were divided by their own mean KCC within the mask for standardization purposes to compute the mean ReHo (mReHo). We conducted a whole-brain voxelwise comparison using FDR-corrected t-tests to evaluate group differences in mReHo between the SA, the NS and the HC. We also assessed the relationship between the SA participants’ mReHo and the HAM-D/HADS scores using multiple regressions. Age, gender, and years of education were used as the covariates. To view the results, we used a T1-weighted MNI template to create the underlying map.

### 2.7. Graph Theoretical Analysis (GTA)

In GTA, we first defined a set of nodes and edges. Using the functional connectivity toolbox (CONN, the Gabrieli Lab., McGovern Institute for Brain Research, MIT, Cambridge, MA, USA), the whole brain was divided into 90 regions of interest (ROIs) (45 per hemisphere) with an automated anatomical labeling (AAL) template, each of which was considered a node [22,23]. The edge represents the brain’s functional connectivity between two nodes. The degree of a node is the number of edges connecting it to the rest of the network, which allows characterizing the edge distribution of all nodes in the network [24].

The resting-state functional image was registered to the T1-weighted image and then to the MNI space. The transformation matrix from the resting space to MNI space was calculated by the transformation matrices created in the two aforementioned register processing steps and was stored for later use. We spatially normalized the resting-state functional images to the AAL template in MNI native space, and the connectivity matrix was obtained after functional connectivity analysis. The functional connectivity matrix was acquired from the functional connectivity toolbox (CONN). Finally, we performed a graph theoretical analysis by using the connectivity matrix.

Network properties analyses were performed using the graph analysis toolbox (GAT, Stanford University School of Medicine, Stanford, CA, USA) [25]. The previous analysis produced a 90 × 90 association connectivity matrix for each individual. The GAT extracted the regional mean time series of each of the 90 ROIs, and partial correlations were used to construct undirected weighted networks. The density range in which a network comparison is meaningful needed to be identified (i.e., the density range in which the networks were not fragmented) [25] before the statistical analyses. After all of the networks were examined, the minimum network density at which no individual network was fragmented was identified as 0.2. The maximum density of the network was determined by the percent of connections present using the most lenient threshold applied, which was 0.5. Next, the networks of the two groups were created at different correlation thresholds, ranging from 0.2 to 0.5, in 0.01 increments. Using graph theoretical analysis to calculate the topological parameters of the brain network, including clustering coefficient (C), normalized clustering coefficient (γ), local efficiency (E_local_), characteristic path length (L), normalized characteristic path length (λ), global efficiency (E_global_), small-worldness (σ), and transitivity. To determine the statistically significant differences between the groups in the network topology and regional network measurements, we manually extracted the area under the curve between 0.2 and 0.5 of the density to calculate the *p* value of the two-sample t-test.

### 2.8. Network-Based Statistical (NBS) Analysis

An NBS analysis was used to identify the significance of any connected subnetworks evident in the set of altered connections found in the healthy controls and the depressed patients with and without suicide attempt. The NBS analysis tried to identify any potentially connected structures formed by an appropriately chosen set of suprathreshold links. The topological extent of any such structure was then used to determine its significance. The test statistic (i.e., primary threshold) computed for each pairwise association was used to construct a set of suprathreshold links [26]. The null distribution of the number of edges was empirically obtained using nonparametric permutation (5000 permutations) to assess the significance of each of the connected edges.

## 3. Results

### 3.1. Participants

Table 1 shows the demographic characteristics. There were significant differences in age, gender, and years of education among the three groups. Therefore, age, gender, and years of education were used as covariates for subsequent analyses.

### 3.2. FC and SCA

**Figure 1:**
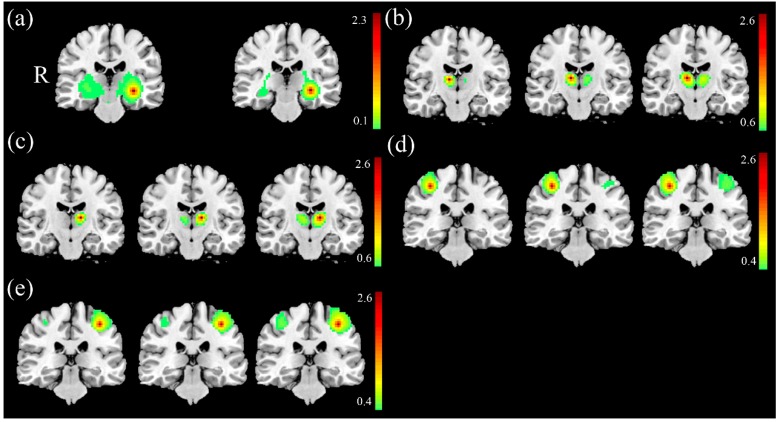
Higher FC of the (**a**) left hippocampus and lower FC of the (**b**) right thalamus and (**c**) left thalamus was found in the SA compared with the NS. Lower FC of the (b) right thalamus, (c) left thalamus, (**d**) right motor cortex, and (**e**) left motor cortex was found in the SA compared with the HC. The lower FC of the (b) right thalamus, (c) left thalamus, (d) right motor cortex, and (e) left motor cortex in the NS compared with the HC.

In FC analysis between the SA and the NS, we found higher FC of the left hippocampus in the SA (Figure 1a) and lower FC of the right thalamus (Figure 1b) and left thalamus (Figure 1c) in the SA compared with the NS (corrected *p* < 0.05). In FC analysis between the SA and the HC, we found lower FC of the right thalamus (Figure 1b), the left thalamus (Figure 1c), the right motor cortex (Figure 1d), and the left motor cortex (Figure 1e) in the SA compared with the HC (corrected *p* < 0.05). In FC analysis between the NS and the HC, we found lower FC of the right thalamus (Figure 1b), the left thalamus (Figure 1c), the right motor cortex (Figure 1d), and the left motor cortex (Figure 1e) in the NS compared with the HC (corrected *p* < 0.05).

### 3.3. VBA of mfALFF and mReHo

**Figure 2:**
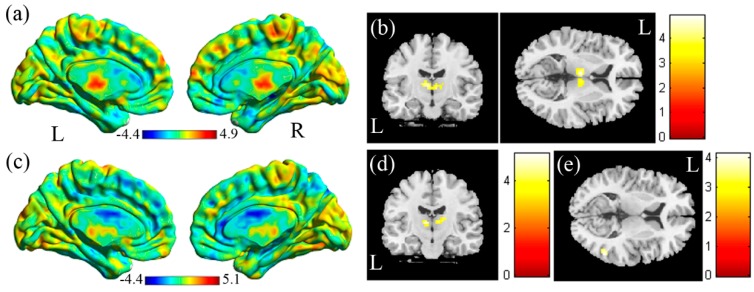
(**a**) Two-sample t-test results of the mfALFF between the SA and HC groups (HC > SA, the color bar represents the t-score). (**b**) Lower mfALFF of the right and left thalamus was found in the SA compared with the HC. (**c**) Two-sample t-test results of mReHo between the SA and the HC groups (HC > SA; the color bar represents the t-score). (**d**) Higher mReHo of the right middle temporal gyrus, and (**e**) lower mReHo of the right and left thalamus was found in the SA compared with the HC.

In the voxel-based analysis (VBA) of the mfALFF between the SA and the HC (Figure 2a, HC > SA), we found lower mfALFF activation of the right and left thalamus in the SA compared with the HC (Figure 2b, corrected *p* < 0.05). In the VBA analysis of mReHo between the SA and the HC (Figure 2c, HC > SA), higher regional homogeneity of the right middle temporal gyrus was found in the SA compared with the HC (Figure 2d, corrected *p* < 0.05). Lower regional homogeneity of the right and left thalamus was found in the SA compared with the HC (Figure 2e, corrected *p* < 0.05).

**Figure 3:**
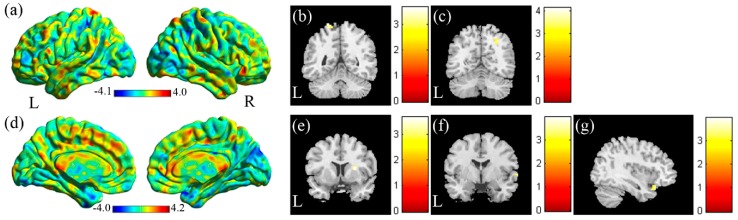
(**a**) Two-sample t-test results of the mfALFF between the SA and NS groups (SA > NS, the color bar represents the t-score). (**b**) Higher mfALFF of the left superior parietal gyrus and (**c**) lower mfALFF of the right angular gyrus was found in the SA compared with the NS. (**d**) Two-sample t-test results of mReHo between the SA and NS groups (SA > NS; the color bar represents the t-score). (**e**) Higher mReHo of the right putamen and (**f**) lower mReHo of the right superior temporal gyrus (STG), and (**g**) right inferior orbital frontal gyrus was found in the SA compared with the NS.

In the VBA analysis of the mfALFF between the SA and the NS (Figure 3a, SA > NS), we found higher mfALFF activation of the left superior parietal gyrus in the SA compared with the NS (Figure 3b, corrected *p* < 0.05) and lower mfALFF activation of the right angular gyrus (Figure 3c, corrected *p* < 0.05). In the VBA analysis of mReHo between the SA and the NS (Figure 3d, SA > NS), we found higher regional homogeneity of the right putamen in the SA compared with the NS (Figure 3e, corrected *p* < 0.05). We also found lower regional homogeneity of the right superior temporal gyrus (STG) (Figure 3f, corrected *p* < 0.05) and right inferior orbital frontal gyrus (Figure 3g, corrected *p* < 0.05) in the SA compared with the NS.

**Figure 4:**
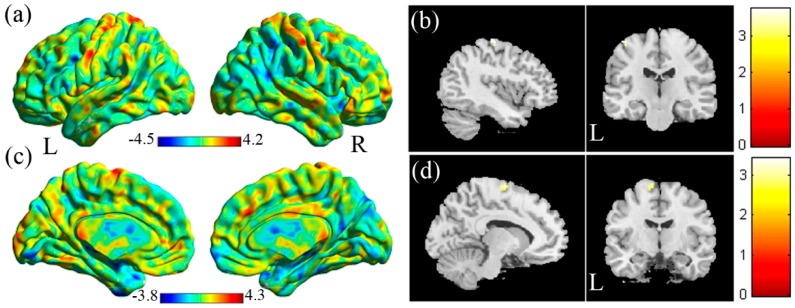
(**a**) Two-sample t-test results of the mfALFF between the HC and NS groups (HC > NS, color bar represents t-score). (**b**) Lower mfALFF of the left precentral gyrus was found in the NS compared with the HC. (**c**) Two-sample t-test results of mReHo between the HC and NS groups (HC > NS; the color bar represents the t-score). (**d**) Lower mReHo of the left supplementary motor area was found in the NS compared with the HC.

In the VBA analysis of the mfALFF between the HC and the NS (Figure 4a, HC > NS), we found lower mfALFF activation of the left precentral gyrus in the NS compared with the HC (Figure 4b, corrected *p* < 0.05). In the VBA analysis of mReHo between the NS and the HC (Figure 4c, HC > NS), lower regional homogeneity of the left supplementary motor area was found in the NS compared with the HC (Figure 4d, corrected *p* < 0.05).

### 3.4. Association between HAM-D, Anxiety/Depression Scores of HADS, and mfALFF/mReHo

**Figure 5:**
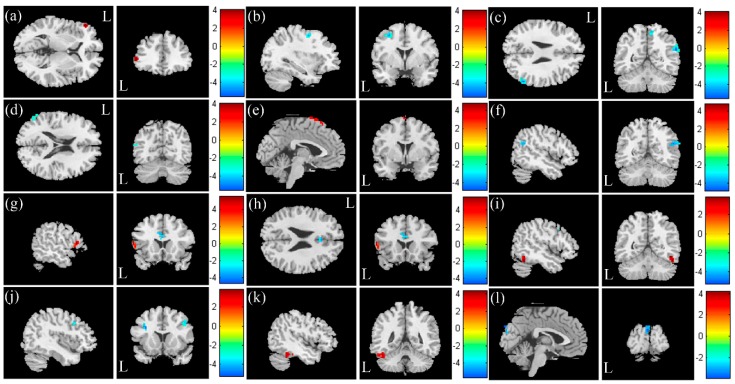
(**a**) Positive correlation between HAM-D and mfALFF in left inferior triangular frontal gyrus. (**b**) Negative correlation between HAM-D and mfALFF in left precentral gyrus, left middle frontal gyrus. (**c**) Negative correlation between HAM-D and mfALFF in right angular gyrus. (**d**) Negative correlation between HAM-D and mfALFF in left middle temporal gyrus. (**e**) Positive correlation between HAM-D and mReHo in left supplementary motor area. (**f**) Negative correlation between HAM-D and mReHo in right superior temporal gyrus and right angular gyyrus. (**g**) Positive correlation between anxiety score and mfALFF in left inferior triangular frontal gyrus. (**h**) Negative correlation between anxiety score and mfALFF in bilateral anterior cingulate cortex. (**i**) Positive correlation between anxiety score and mReHo in right inferior temporal gyrus. (**j**) Negative correlation between anxiety score and mReHo in right middle frontal gyrus. (**k**) Positive correlation between depression score and mfALFF in left inferior temporal gyrus, left fusiform. (**l**) Negative correlation between depression score and mReHo in left cuneus gyrus.

In a multiple regression analysis (Figure 5), regarding the relationship between HAM-D scores and the mfALFF, a positive correlation in the left inferior triangular frontal gyrus (Figure 5a, *p* < 0.05) was found, and a negative correlation in the left precentral gyrus, left middle frontal gyrus (Figure 5b, *p* < 0.05), right angular gyrus (Figure 5c, *p* < 0.05), and left middle temporal gyrus (Figure 5d, *p* < 0.05) were found. A positive correlation between HAM-D scores and mReHo in the left supplementary motor area (Figure 5e, *p* < 0.05) was found, and a negative correlation in the right superior temporal gyrus and right angular gyrus (Figure 5f, *p* < 0.05) was found. A positive correlation between anxiety scores and the mfALFF in the left inferior triangular frontal gyrus (Figure 5g, *p* < 0.05) was found, and a negative correlation in the bilateral anterior cingulate cortex (Figure 5h, *p* < 0.05) was found. A positive correlation between anxiety scores and mReHo in the right inferior temporal gyrus (Figure 5i, *p* < 0.05) was found, and a negative correlation in the right middle frontal gyrus (Figure 5j, *p* < 0.05) was found. A positive correlation between depression scores and the mfALFF in the left inferior temporal gyrus and left fusiform (Figure 5k, *p* < 0.05) were found. A negative correlation between depression scores and mReHo in the left cuneus gyrus (Figure 5l, *p* < 0.05) was found.

### 3.5. GTA

**Figure 6:**
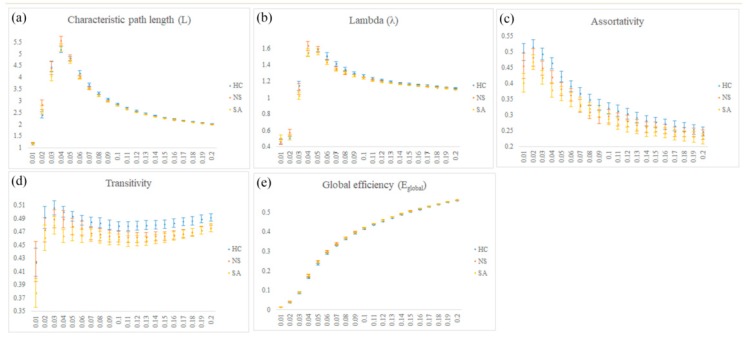
The topological parameters including (**a**) characteristic path length, (**b**) normalized characteristic path length (λ), (**c**) assortativity, (**d**) transitivity, and (**e**) global efficiency among the three groups, i.e., the SA, the NS, and the HC.

In the GTA among the three groups, i.e., SA, NS, and HC, we found a significant tendency in the characteristic path length (Figure 6a, corrected *p* < 0.05), normalized characteristic path length (λ) (Figure 6b, corrected *p* < 0.05), global efficiency (Figure 6c, corrected *p* < 0.05), assortativity (Figure 6d, corrected *p* < 0.05) and transitivity (Figure 6e, corrected *p* < 0.05). However, no significant tendency was found in the clustering coefficient, normalized clustering coefficient (γ), local efficiency, and small-worldness (σ). Although all participants maintained the small-worldness functional brain network according to the σ calculation, the network was more like a random network in the SA.

### 3.6. NBS Analysis

**Figure 7:**
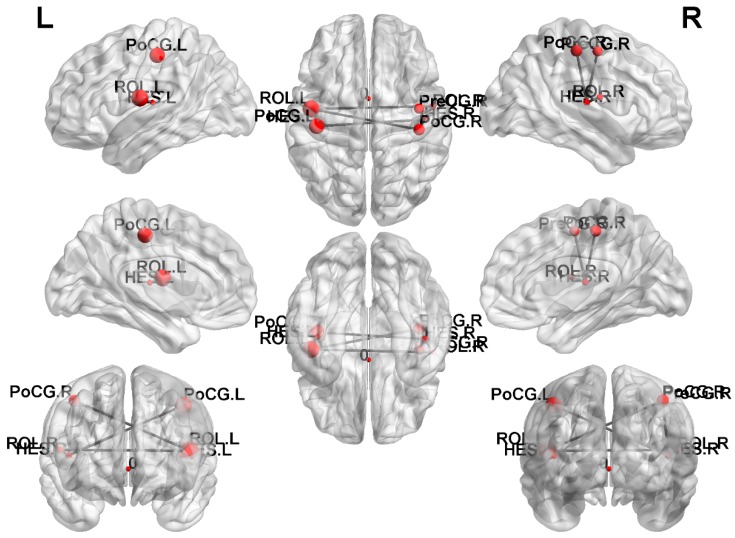
The NBS result showed the disrupted subnetwork in the SA compared with the HC (HC > SA).

In the NBS analysis, we compared the edges of the brain networks between the SA and the HC groups. One subnetwork showed more edges in the HC compared with the SA (Figure 7, corrected *p* < 0.05), including the connections from the left rolandic operculum to the right rolandic operculum, postcentral gyrus, and the left Heschl’s gyrus; from the right Heschl’s gyrus to the right precentral gyrus, postcentral gyrus, rolandic operculum, and the left postcentral gyrus. We also compared the edges of the brain networks between the SA and the NS and the NS and HC; however, no significant results were found.

## 4. Discussion

Our results demonstrated that widespread, but disrupted, network changes in brain functional networks and their interconnectivity was associated in depressive patients with suicidal attempt. Our results suggest that the neural basis underlying the psychopathology of attempted suicide in depressive patients involves multiple brain functional networks and their interaction. In the SCA analysis, we found significant results among the three groups in the hippocampus, the hypothalamus, and the motor cortex. When the left hippocampus was activated, the activation of the right hippocampus of the SA group was greater than that in the NS group. In a previous study [27], it was found that the hippocampal gyrus volume of controls was greater than major depressive disorder (MDD). We speculate that when the hippocampal gyrus volume on one side decreases, the activity of the contralateral brain region may increase to compensate for the functional integrity. In another study [14], it was found that the mReHo value of the left hippocampus of the SA was smaller than that of the HC, and the regional homogeneity around the left hippocampus of the SA group was lower, which may be related to the volume reduction. The functional connectivity between the left and right thalamus was also significant between each group, i.e., HC is greater than NS and greater than SA. In other studies, it was found that the mReHo of the left hypothalamus of HC was greater than that of MDD [28], and the zALFF value of the HC left hypothalamus was greater than that of the SA and NS [12]. When the activity on one side increases, it may increase the functional connection with the contralateral brain area.

In the VBA, t-tests were compared between each group. In the comparison between the SA and HC, both the mfALFF and mReHo results showed that both sides of the HC were larger than SA, which was consistent with the results of other studies [12,28]. In still other studies [29,30,31,32], it was found that the gray matter volume of the hypothalamus was reduced, and that the decrease in volume may have led to a decrease in its activity, which was similar to our the mfALFF results. The decrease of mReHo in the bilateral thalamus of the SA indicates that the similarity between the surrounding voxels is low, and the activation range is small.

In our mReHo results, we found that the mReHo value in the right middle temporal gyrus of the SA was higher than that of the HC. In a previous study [10], it was observed that the functional connectivity between the right amygdala and the left middle temporal gyrus was greater than that of the NS. Another study found that the zALFF value in the middle temporal gyrus of the SA was greater than that of the NS and HC [12] and found that the functional connectivity between the SA and NS in the right middle temporal gyrus was greater than the HC [11]. All of these results were similar to our results, which may indicate abnormal activation of the middle temporal gyrus in patients with MDD and provide partial evidence that abnormal activation of the middle temporal gyrus is associated with suicidal behavior.

In our VBA results of the SA and NS groups, we found that the mfALFF of the right angular gyrus in the SA was smaller than in the NS. A previous study found that the gray matter volume of the left angular gyrus of SA was small [33]. In the mReHo analysis [14], it was found that mReHo of the right angular gyrus in the SA was smaller than that in the HC. In the ALFF analysis [13], only the left angular gyrus of the HC group was less than the SA and NS group, and there was no significant finding in the comparison between the NS and SA groups. However, another study found that the zALFF value of the left angular gyrus in the SA was greater than in the NS and that the HC was greater than NS [12]. In our mReHo results of the SA and NS, we found that the mReHo value of the right putamen of SA was greater than that of NS; however, these VBM studies found that the putamen and the gray matter volume of the SA was less than HC [34]. In our mReHo results of the SA and NS, it was also found that the mReHo of the right superior temporal gyrus in the SA was lower than that in the NS. In the seed-based analysis [35], it was found that the functional connectivity of MDD patients’ subgenual anterior cingulate cortex (ACC)-based neural network was smaller than HC, and the network included the superior temporal gyrus. However, the ALFF results found that the ALFF/zALFF values of the superior temporal gyrus of the SA were greater than those of the NS and HC [12,13]. Finally, we found that the mReHo of the inferior orbitofrontal gyrus in the SA was smaller than in the NS, which was consistent with a previous study [14]. The mReHo of the left supraorbital inferior frontal gyrus in the SA was smaller than in the HC. This finding may indicate that the inferior orbitofrontal gyrus can be used to distinguish between the HC and SA and may also be used between the SA and NS.

In the multiple regression analysis, the correlation analysis between the mfALFF/mReHo and the HAM-D, anxiety/depression scores of HADS, in the depressed patients with a history of suicide attempt was performed. When a positive correlation was present, higher scores indicated higher activity of the brain region (SA > HC). When a negative correlation was present, lower scores indicated higher activity of the brain region (HC > SA). In the correlation between the mfALFF and HAM-D, we found a positive correlation in the left inferior triangular frontal gyrus in the SA, and they were negatively correlated in the left precentral gyrus and middle frontal gyrus. Previous studies found that the fALFF value in the right middle frontal gyrus of MDD patients was smaller than that of HC [36] and found that the ALFF value in the ventral middle frontal gyrus was smaller in the SA than in the NS [13] and that the zALFF value of the left middle frontal gyrus in the SA was less than that of the HC [12]. All of the results were similar to our correlation analysis, and the ALFF of the middle frontal gyrus may be used to distinguish between the SA and NS. We found a negative correlation between HAM-D and the right angular gyrus of the SA, and the VBM study found that the gray matter volume of the left angular gyrus was smaller than that of NS [33]. Other studies found that the mReHo value in the right angular gyrus of the SA was less than the HC [14] and found that the left angular gyrus of the NS had a zALFF value less than the SA and HC [12]. This result may indicate that the angular gyrus can be used to distinguish between the SA and NS and to distinguish between the NS and HC.

Regarding the correlations between the mReHo and the HAM-D scores, we found a positive correlation in the left supplementary motor area of the SA, and negative correlations in the right superior temporal gyrus and angular gyrus. Previous studies found that the functional connectivity of the superior temporal gyrus of MDD patients was less than that of HC [35], and VBM analysis found that the left angular gyrus volume of the SA was smaller than that of the NS [33]. Another study found that the mReHo value in the right angular gyrus of SA was less than the HC [14]. These results were similar to our results, and the activity of the superior temporal gyrus and angular gyrus of patients with depression was lower than that of normal controls. However, another study found that the ALFF value of the right superior temporal gyrus in SA was greater than in NS and HC, and the ALFF value of the left angular gyrus in HC is smaller than in SA and NS [13]. Cao’s study found that the mReHo value of the right angular gyrus in SA was less than HC [14] and found that the zALFF value of the right middle temporal gyrus in SA was greater than that of NS and HC, and the zALFF value of the left angular gyrus in NS was smaller than that of the SA and HC [12]. These findings are somewhat different from our results, and we speculate that the superior temporal gyrus and angular gyrus also have a certain relationship with depression, but the mechanism is still unclear.

Regarding the correlations between the mfALFF and the anxiety scores, we found a positive correlation in the left inferior frontal gyrus of the SA, and a negative correlation in the bilateral anterior cingulate cortex (ACC). One study found the functional connectivity of the left inferior frontal gyrus and subgenual ACC in MDD patients was less than HC [35], and another study found that the ALFF value in the left ACC of SA and NS was greater than that in HC [13]. Regarding the correlation between mReHo and anxiety scores, there was a positive correlation in the right inferior temporal gyrus of the SA, and a negative correlation in the right middle frontal gyrus. In previous studies, the fALFF value of the right middle frontal gyrus in MDD patients was found to be less than that in HC [36] the ALFF value of the ventral middle frontal gyrus of NS was greater than that of SA and HC [13] and the mReHo value in the bilateral middle frontal gyrus of SA was less than HC [14]. Other studies found that the zALFF of the left middle frontal gyrus of NS was greater than that of HC and SA [12] and found that the functional connectivity in the left middle frontal gyrus of SA and NS was greater than that of HC [11]. Most of the studies found lower functional activity in SA than that in NS and HC; therefore, we speculated that the change of the functional activity in the middle frontal gyrus was related to the presence of suicide attempt.

In the graph theoretical analysis, we found that the characteristic path length, lambda, assortativity, and transitivity of the HC were greater than those of the SA, and the global efficiency of the HC was less than the NS, which was significant. The characteristic path length represents the ability to quantify the transition between brain regions. The smaller the value is, the better the global integration in the brain is. Lambda is a normalized path length for the normalization of 100 random brains. Like the characteristic path length, the smaller the value is, the better the brain integration ability is. Our results showed that the SA had a slightly better global integration ability. Assortativity is the correlation between the link between two nodes and degree. Positive assortativity means that nodes with high degree are easy to connect with each other. Transitivity is a classic transform of the clustering coefficient, which is used to quantify the interconnection between local networks. The larger the value, the better the brain segmentation ability. It is speculated that the HC brain has a slight segmentation ability. In the graph theoretical analysis of diffusion tensor imaging (DTI) [37], it was found that SA had a shorter characteristic path length, similar to our findings. Finally, the results of the NBS study revealed that the number of connections between brain regions in the HC was greater than in the SA, and the results were significant. In the DTI study, they also found that the number of connections between brain regions in HC was greater than those in MDD patients [38]. Although the location of the brain regions is not the same, overall, the number of connections in the normal human brain network is more than that in patients with depression. 

## 5. Conclusions

In this SCA, we found altered FC in some brain regions of the SA group compared with the NS or HC groups, especially in the hippocampus and thalamus. In the VBA, the mfALFF and mReHo results showed different activation and regional homogeneity of the temporal lobe and several brain regions in the SA compared with the NS and HC. We also found that aspects of activity in some brain areas correlated with the HAM-D scores, anxiety scores, and depression scores, especially in the frontal and temporal lobes. We concluded that the brain function of people with major depressive disorders with and without suicide attempts changed compared relative to healthy participants. GTA and NBS analyses revealed different topological organization, as well as slightly better global integration and worse local segregation of the brain network (i.e., more like a random network) in depressed participants compared with healthy participants. We suggest that depressed patients with or without suicide attempt may affect their brain functional connectivity. Further studies are warranted to include larger sample size of suicide attempters to explore the association between different levels of severity of suicide attempt and brain image, such as the level of suicide intent or lethality of suicide methods. 

## Figures and Tables

**Table 1:** Summary of characteristics of demographic data and neuropsychological tests.

Characteristics	Depressive Patients with Suicide Attempt (SA, *n* = 33)		Depressive Patients Without Suicide Attempt (NS, *n* = 32)		Healthy Controls (HC, *n* = 44)		ANOVA	A (SA vs. NS)	B (SA vs. HC)	C (NS vs. HC)
	Mean of Counts	SD	Mean of Counts	SD	Mean of Counts	SD	*p*-value	Corrected *p*-Value**		
Age	43.55	7.79	47.88	9.00	41.98	9.27	0.017	0.046	0.43	0.008
Gender (M/F)	3/30	N/A	13/19	N/A	7/37	N/A	N/A	0.000482	0.378	0.015
Education (years)	11.52	2.61	12.84	2.87	13.53	3.15	0.014	0.059	0.003	0.331
HAM-D	19.30	8.04	15.16	7.13	5.14	5.79	<0.05	<0.05	<0.05	<0.05
Anxiety of HADS	11.97	5.45	7.22	4.15	4.79	3.75	<0.05	<0.05	<0.05	<0.05
Depression of HADS	11.85	4.60	6.88	4.69	3.86	3.15	<0.05	<0.05	<0.05	<0.05

SD: Standard deviation; HAM-D: Hamilton depression rating scale; HADS: Hospital Anxiety and Depression Scale; N/A: not applicable; **corrected *p*-value: <0.05 indicating significant difference; A: T-test between depressive patients with suicide attempt and depressive patients without suicide attempt; B: T-test between depressive patients with suicide attempt and healthy controls; C: T-test between depressive patients without suicide attempt and healthy controls; HC, healthy controls; SA, suicide attempt.
